# Effectiveness of Adjuvant Semaglutide Following Bariatric Metabolic Surgery

**DOI:** 10.1007/s11695-025-07703-0

**Published:** 2025-02-21

**Authors:** Jorgen Ferguson, Oliver Fisher, Michael Talbot, Georgia Rigas

**Affiliations:** UNSW Sydney, Sydney, Australia

## Abstract

**Background:**

Obesity is a relapsing condition and response to anti-obesity therapies appears to be normally distributed. Therefore, some patients undergoing metabolic bariatric surgery (MBS) will demonstrate a partial response to therapy. When prescribing therapies to patients living with obesity (PwO) the median total weight loss (TWL) gives a good indication of the likely utility of prescription for that individual. GLP-1 agonists (GLP1a) offer patients a reasonable prospect of clinically significant weight loss even if they have been previously treated with MBS.

**Methods:**

A retrospective review of prospectively collected data in a single bariatric clinic was performed. Patients with insufficient weight loss at any time point were offered semaglutide therapy with doses titrated depending on response to treatment, tolerability, availability and affordability. Duration of therapy, highest dose tolerated, anthropometric measures and reported side effects were recorded. Reasons for discontinuation were noted where possible; however, discontinuation due to medication unavailability was not reliably captured in the dataset.

**Results:**

The median dose tolerated was 1 mg s/c per week, and 78% tolerated ≤ 1 mg as the maximum achieved dose. The median TWL was 7.5% and side effects were uncommon. Most patients took therapy for > 6 months, but continued therapy > 1 year was uncommon.

**Conclusion:**

Overall ‘real-world’ utility of semaglutide after MBS may potentially be hampered by supply and cost issues more than issues associated with effectiveness or side effect profile.

## Introduction

The suboptimal initial response to MBS and/or worsening of a significant obesity complication that occurs after an initially adequate post-operative clinical response are not an uncommon occurrence [1]. The reasons for this are not fully understood; however, genetics [2] and hormonal mechanisms [3] have been implicated. The incretin effect of GLP-1 receptor agonists (GLP1a) in the medical management of obesity is well documented [4, 5] by stimulating insulin release, suppressing glucagon secretion, reducing gastric emptying and promoting increased satiety [6]. The efficacy and tolerability of liraglutide (daily s/c GLP1a) have been reported elsewhere [7–9] and have been shown to improve weight loss outcomes in that where satisfactory weight loss following surgery has not been achieved [10]. Within this class, semaglutide represents a unique GLP1a, with both subcutaneous (s/c) and oral routes of administration available [11]. The Therapeutic Good Administration (TGA) has approved the use of high dose semaglutide (2.4 mg) for management of obesity in Australia; however, the oral equivalent will not be available for the foreseeable future.

Several randomised trials (STEP) [12–15] have compared outcomes following weekly subcutaneous administration of semaglutide to both placebo and lifestyle modification in patients mostly without diabetes with overweight/ obesity. The STEP trials showed that semaglutide resulted in significant weight loss in patients with and without diabetes and these effects have been sustained out to 2 years.

Similar results have been borne out in several meta-analyses. Once weekly semaglutide s/c injection results in a pooled reduction in total body weight of 10–12.57% and reductions in both BMI and waist circumference when compared to placebo [16, 17]. High dose s/c semaglutide is also more effective than low dose s/c semaglutide, oral semaglutide and other GLP1a’s for weight loss [18]. With this in mind, we aimed to analyse weight loss outcomes for semaglutide at the maximum tolerated dose in a cohort of post-surgical bariatric patients.

## Methods

We performed a combined retrospective and prospective single centre, cohort study of patients who received semaglutide to assist in weight loss at a single bariatric centre. Post-MBS patients were offered GLP1a pharmacotherapy (s/c semaglutide in different dosing regimens) if reporting inadequate weight loss or significant weight regain at follow-up. Inadequate weight loss was defined depending on the patient’s previous surgery and the duration of time since surgery. Patients had inadequate weight loss defined as BMI > 35 (all patients), < 25% TWL (LSG, RYGBP), < 20% TWL (LAGB) and in patients who self-identified as weight stable after surgery, who did not achieve their own pre-operatively defined health or weight goals. Significant weight regain beyond this time was defined as weight regain of > 5 kg from nadir. Patients with inadequate weight loss were therefore assessed in 1 of 3 groups: insufficient weight loss following bariatric surgery (*partial responder*), those with insufficient weight loss and associated weight regain (*partial responder* + *weight regain*) or those experiencing sufficient initial weight loss with subsequent weight regain (*weight regain*).

Subgroup analysis was performed according to the type of bariatric surgery performed (roux-en-y gastric bypass (RYGB), sleeve gastrectomy (SG) and adjustable gastric band (AGB)) and according to the nature of the surgery i.e. whether the procedure was a primary or revisional case with revisional cases including patients whose primary procedures were performed either within or outside of our centre. Patients were followed up in the office or via telephone. Patients were excluded from analysis if they could not be contacted or declined to participate.

Data were collected regarding change in body weight and BMI, following semaglutide therapy. Secondary outcomes included therapy duration and associated side effects with maximum tolerated doses of semaglutide. Patients were offered escalating doses of semaglutide (0.25, 0.5, 1.0, 1.25, 1.5 and 2.0 mg depending on tolerance and affordability) and dosing was assessed at 3 monthly intervals. Data are presented as median values with interquartile ranges unless otherwise specified. Statistical analyses were performed using paired t-test to assess outcomes before and after pharmacotherapy. Statistical significance was conveyed with an alpha value of < 0.05. All analyses were performed using R Statistical Programming.

## Results

**Fig. 1:**
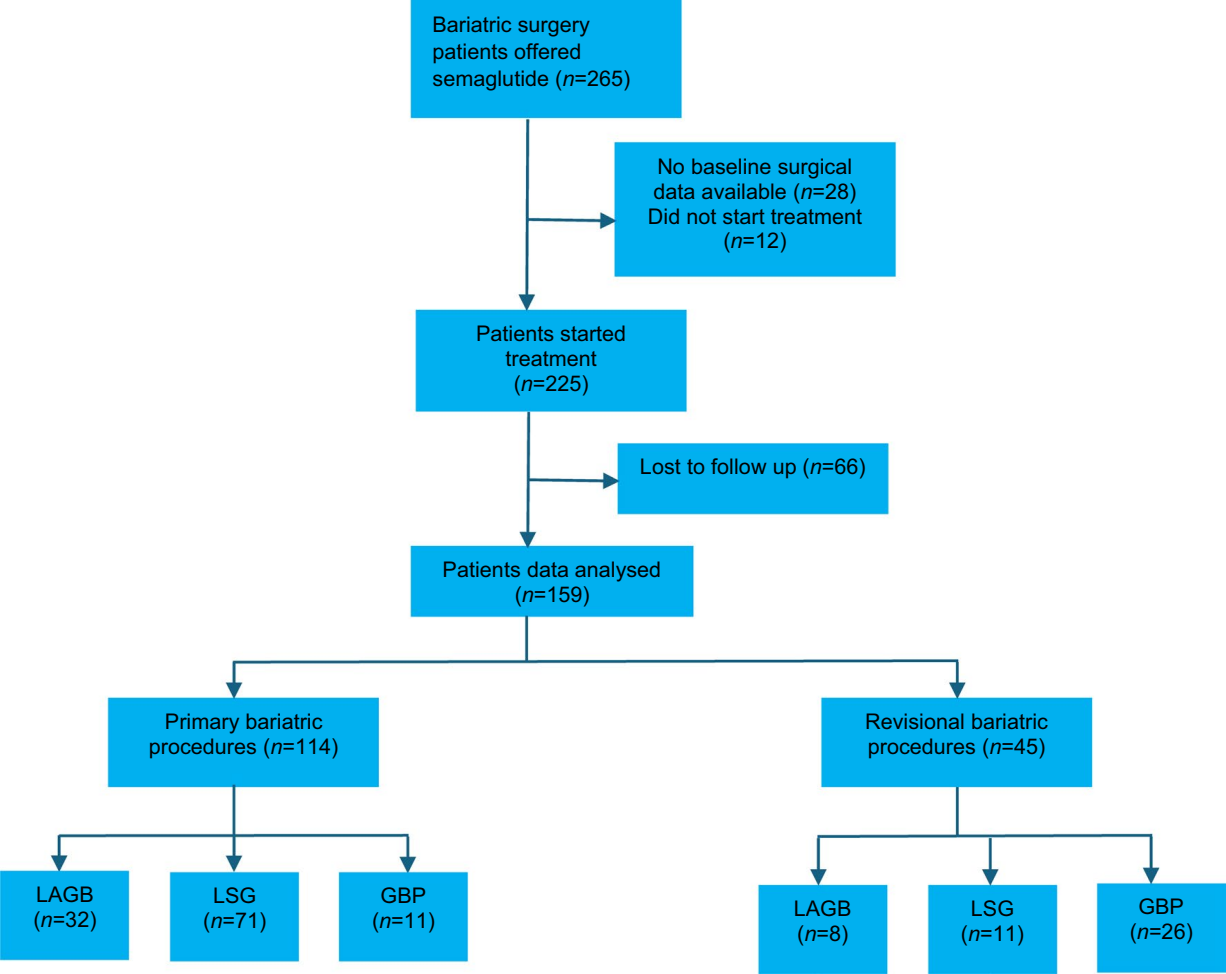
Patient selection

From July 2020 to December 2021 a total of 265 patients were offered adjuvant pharmacotherapy with semaglutide. Following exclusion of patients that agreed to participate in the study but did not commence treatment, patients with inadequate baseline surgical data and patients lost to follow up, 159 patients were left for analysis (Fig. 1). These patients had undergone either primary or revisional bariatric surgery between January 2002 and December 2021.

Baseline demographic data and patient surgical anatomy are presented in Table 1 and Table 2. respectively. The majority of the patients were female (129/159 (81.1%)) with a mean age of 40.9 years at most recent follow-up. Diabetes and pre-diabetes were evident in 84/159 (59.1%) of patients. Other comorbidities are shown in Table 1.

**Table 1:** Baseline demographic data

	Number of patients
Female (%)	129 (81.1)
Significant comorbidities	
N	14 (8.8)
Y	145 (91.2)
Comorbidities	
HT (%)	66 (41.5)
T2DM (%)	15 (9.4)
Insulin resistance (%)	79 (49.7)
OSA (%)	31 (19.5)
Dyslipidaemia (%)	84 (52.8)
Thyroid disease (%)	28 (17.6)
Mental health (%)	74 (46.5)
Weight at commencement of semaglutide (kg) (range)	96.5 (84.05, 112.55)

**Table 2:** Patient subgroups prior to semaglutide therapy

	Partial responder	Partial responder + weight regain	Weight regain
Primary surgery (%)			
AGB	1 (1.0)	3 (1.9)	28 (17.6)
SG	17 (10.7)	14 (8.8)	40 (25.2)
RYGB	5 (3.1)	1 (1.0)	5 (3.1)
Revisional surgery (%)			
AGB	0	0	8 (5.0)
SG	1 (1.0)	2 (1.3)	8 (5.0)
RYGB	8 (5.0)	4 (2.5)	14 (8.8)

Laparoscopic sleeve gastrectomy (LSG) was the most commonly performed procedure (*n* = 82, (51.6%)) followed by laparoscopic adjustable gastric band (LAGB) (*n* = 40, (25.2%)) then gastric bypass (GBP), *n* = 37, (23.3%). The majority of procedures performed were primary procedures (*n* = 114) with gastric bypass being the most commonly performed revisional procedure (*n* = 26/45). The median preoperative BMI was 43.2 kg/m^2^ (IQR 40–47) with patients achieving a nadir weight of 79.6 kg (73–93). Immediately prior to the commencement of semaglutide, the median weight had increased to 94.3 kg (81–104). The majority of patients were commenced on semaglutide for ‘weight regain’ (*n* = 103, (64.8%)) and ‘weight regain’ was also the most common reason for commencing therapy in both primary and revisional cases.

**Fig. 2:**
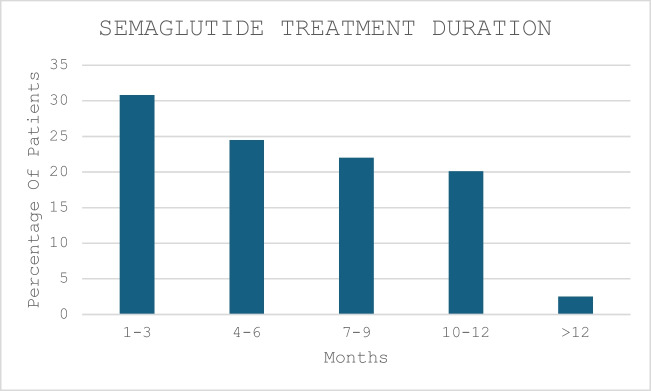
Duration of semaglutide therapy

**Fig. 3:**
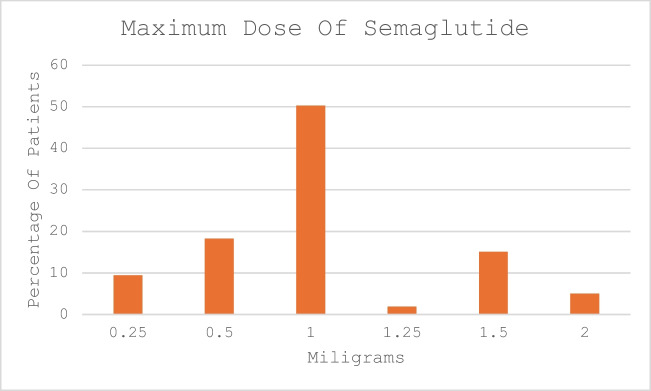
Maximum semaglutide dose achieved

The pooled data showed a median reduction in weight of 7.5 kg (4–13) and a median decrease in BMI of 2.8 kg/m^2^ (2–5) following semaglutide. This corresponded to a percentage total weight loss (%TWL) of 7.5%. For patients taking 0.5 mg and 1.0 mg doses of semaglutide, the range of %TWL varied between 10 and 14%. Therapy duration and dosing are shown in Figs. 2 and 3, respectively. More than half of the cohort continued therapy beyond 6 months (the median duration was 6 months) with only 4 patients extending therapy beyond 12 months. As expected, continued adherence with therapy correlated with a greater degree of weight loss. The greatest difference in weight loss occurred between months 9 and 12; however, this effect plateaued at 12 months (although these results were seen in a smaller cohort). The median maximum dose of semaglutide was 1 mg (0.5, 1.0) and this was achieved in 50% of the cohort (78% of the cohort took doses equal to or less than 1 mg). Despite this, there was no association between the duration of treatment and the dose administered. There was no difference in the maximum achieved dose of semaglutide between primary and revision cases and the weight loss achieved for both groups was not significantly different (*p* = 0.4). Men took a higher dose of semaglutide compared to women (Fig. 3).

**Fig. 4:**
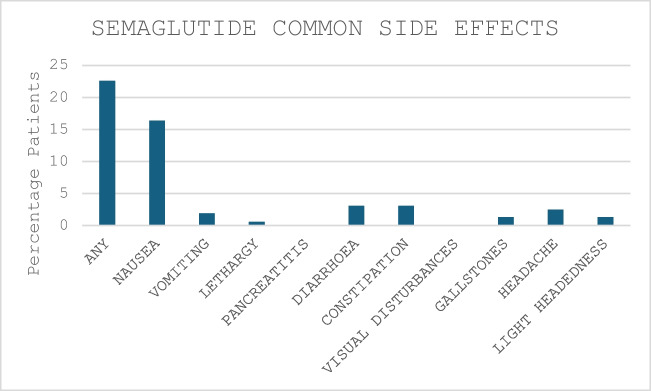
Reported side effects following semaglutide therapy

At the maximum achieved dose of semaglutide, 36 patients (22.6%) reported one or more side effects (Fig. 4). As expected, the most common side effects were nausea, change in bowel function and headache.

## Discussion

Our study has demonstrated that administration of a GLP1a to patients with refractory obesity following bariatric surgery results in additional weight loss. The effect of a GLP1a on weight loss seems to be independent of the type of bariatric procedure and is equally efficacious in both primary and revisional cases.

Our percentage weight loss outcomes are similar to those previously reported. The effects of GLP1a on weight loss have been known for almost 2 decades. In one of the earliest larger systematic reviews [19], GLP1a (exenatide and liraglutide) compliance resulted in superior weight loss in patients without diabetes when compared to those with diabetes. However, in that study, adverse events were more prevalent with increasing doses of a GLP1a. In their recent meta-analysis and systematic review, Vosoughi et al. [18] reported directly on their outcomes with various analogues of GLP1a’s at different doses. They found that subcutaneous semaglutide 2.4 mg weekly (9.9 kg weight loss) was superior to other GLP1a’s, including doses of semaglutide < 2.4 mg weekly (4.3 kg weight loss). However, in our study, we found adequate weight loss when only using 1 mg s/c weekly, which has significant cost-saving implications for patients.

There have been a few small prospective and retrospective studies looking at pharmacotherapy for weight regain following bariatric surgery. The largest published restrospective study to date was performed by Wharton et al. [8] in which they prescribed 3.0 mg of liraglutide to 117 patients with weight regain at almost 8 years after surgery. These patients achieved a mean total body weight loss of 5.5% of their ‘regain weight’ following a mean of 7 months of therapy. Significant weight loss was seen early on and was maintained out to 12 months in those that were compliant, and weight loss was significantly less pronounced in those patients that discontinued liraglutide prematurely. Jensen et al. [20] looked at weight loss outcomes in 50 patients receiving 6 months of either liraglutide or semaglutide for weight regain following bariatric surgery. The majority of patients received either subcutaneous liraglutide or subcutaneous semaglutide in equal distribution and significant weight loss was seen after 6 months of therapy. Our group has also previously reported on the outcomes of liraglutide whereby post-bariatric surgery patients lost a median TWL of 5.3% but discontinued, mainly due to cost [9].

Our study demonstrates that effective weight loss is possible at doses of GLP1a that are lower than previously reported. The average cost of a monthly semaglutide injection in Australia is about $42 for a Pharmaceutical Benefits Scheme (PBS) prescription (management of diabetes) or $150 per month for a 1.0 mg private prescription (management of weight loss). Currently in Australia, doses of 0.5 mg and 1.0 mg are approved under the PBS for the management of T2DM. The higher doses of semaglutide seen in the STEP trials (2.4 mg) are approved for use in the management of obesity elsewhere under the trade name Wegovy. Wegovy is currently approximately 1.5 times more expensive than semaglutide when prescribed privately.

We have shown we can achieve satisfactory weight loss outcomes in refractory obesity following surgery at lower doses, and that patients select a ‘cost-effective’ dose for themselves with a focus on prolonged therapy rather than escalated therapy. Fifty percent of our cohort maintained a dose of semaglutide of 1.0 mg weekly, with the majority of patients taking a maximum dose of 0.5 or 1.0 mg. In fact, 78% of the cohort in the current study achieved weight loss at doses of 1.0 mg or less. We had reasonable follow-up for all doses of semaglutide up to 7 months; however, beyond 7 months, there was a significant drop off in continuation of therapy. We observed that for each dose of semaglutide, even with the small number of patients continuing therapy beyond 7 months, there was a trend to achieve greater weight loss with continued semaglutide (see supplementary). As side effects only effected 22% of participants, we presume that cessation of semaglutide was due to supply issues where discontinuation due to unavailability may have contributed more than this retrospective analysis was able to pick up. GLP1a have been shown to cause common side effects such as nausea, vomiting, gastrointestinal upset and delayed gastric emptying [21–23]. Our study demonstrated that the side effect profile of semaglutide was less than previously reported, with only 22% of patients reporting common side effects. The lower dose has likely translated into a lower incidence of side effects in our study compared to other studies [12] and that semaglutide-associated gastroparesis seems to not afflict patients who have undergone a RYGB [24].

## Conclusion

This cohort study demonstrates that semaglutide at lower doses compared to previously published is a feasible and cost-effective means of achieving weight loss for refractory obesity in post-operative bariatric patients. The reasons for non-dose escalation no-doubt varied from patient to patient and likely included cost, reduced availability of repeat prescriptions a desire to avoid side effects but also the residual metabolic effects of their surgery which may well have led to adequate appetite suppression at lower doses than what are required in patients with normal anatomy and GI physiology.

## Data Availability

No datasets were generated or analysed during the current study.
